# Exfoliation and Reassembly Routes to a Ge/RuO_2_ Nanocomposite as an Anode for Advanced Lithium-Ion Batteries

**DOI:** 10.3390/ijms231911766

**Published:** 2022-10-04

**Authors:** Jeong-Hun Jang, Minseop Lee, Ji-Hye Koo, Seung-Min Paek

**Affiliations:** Department of Chemistry, Kyungpook National University, Daegu 41566, Korea

**Keywords:** germanium, ruthenium oxide, anode material, lithium-ion battery

## Abstract

Ge/RuO_2_ nanocomposites were successfully fabricated as anode materials for lithium-ion batteries using RuO_2_ nanosheets and Ge/GeO_2_ nanoparticles (NPs). X-ray diffraction (XRD) and X-ray absorption spectroscopy (XAS) analyses showed that elemental Ge nanoparticles were distributed onto the rutile-type RuO_2_. Transmission electron microscopy images showed well-dispersed Ge nanoparticles embedded in rutile-type RuO_2_. The Ge/RuO_2_ nanocomposite maintained higher discharge capacities (471 mA h g^−1^) after the 90th cycle at 0.1 A g^−1^ than that (211 mA h g^−1^) of Ge/GeO_2_ nanoparticles. The Ge/RuO_2_ nanocomposite exhibited a higher capacity retention than Ge/GeO_2_ NPs. These results suggest that the well-dispersed Ge nanoparticles within RuO_2_ matrices enhance the cycle stability and capacity retention of the anode material.

## 1. Introduction

The growing demand for energy-storage devices has triggered extensive research on the development of high-performance metal ion batteries, especially lithium-ion batteries [1,2,3,4,5]. In this regard, the development of next-generation lithium-ion battery (LIB) anode materials is driven by the limited theoretical capacity (372 mA h g^−1^) of graphite, which is a commonly used anode material [6].

Most recent research for obtaining LIBs with enhanced energy density has focused on fabricating alternative anode materials based on group XIV elements and transition metals [7,8]. Germanium is a potential anode material with high theoretical capacity, thermal stability, and good Li-ion conductivity [7,8,9,10,11]. Unfortunately, the large volume changes of Ge-based materials during discharge/charge lead to cracking and pulverization, which are major factors inducing capacity fading in LIBs [12]. Additionally, the oxides of Ru and Ge undergo irreversible conversion reaction with Li to form LiO_2_, which restricts the reversible discharge/charge of the electrodes [13,14]. These defects degrade Li-storage capacities and cycling stabilities. To overcome these drawbacks, various strategies have been developed to fabricate Ge/GeO_2_ and RuO_2_ composites [10,15,16,17,18,19]. The fabrication of oxide-free metallic nanoparticles for anode materials is particularly desirable. Typically, the GeO_2_ component of the Ge/GeO_2_ composite plays an important role in buffering the volume expansion caused by the alloying reaction between Ge and Li. However, the low electrical conductivity of GeO_2_ leads to a decrease in the discharge capacity of the Ge/GeO_2_ composite. For this reason, dispersing Ge nanoparticles in conductive materials, such as carbon-based materials, is recognized as an effective approach for enhancing the electrochemical performance of Ge nanoparticles [20]. Host materials can withstand the volume expansion of Ge and improve the cycling performance of GeO_2_-free Ge nanoparticles. In this regard, the structural engineering of anode materials has been studied for achieving improved capacity retention. For example, TiO_2_, SnO_2_, and RuO_2_ nanosheets (NSs) comprising unique structures, such as flower-like or hollow spheres, exhibit enhanced electrochemical properties [21,22]. In particular, RuO_2_ possesses good chemical stability, high electrical conductivity, and high discharge capacity of 1130 mAh/g [22,23]. These structural and electrochemical advantages render RuO_2_ a good candidate as a matrix material for incorporated anode materials. Hence, well-dispersed Ge nanoparticles in structurally modified RuO_2_ nanosheets are expected to achieve high cycling performance and capacity retention in LIB anodes.

**Figure 1:**
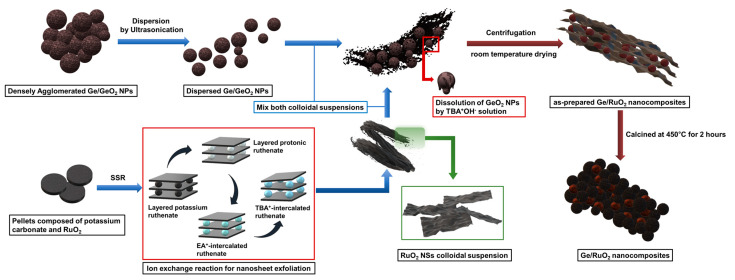
Schematic of synthesis of Ge/RuO_2_ nanocomposites.

In this study, we incorporated Ge nanoparticles (NPs) into matrices of layered RuO_2_ via successive exfoliation and reassembly processes as shown in Figure 1. Notably, nanosized Ge nanoparticles can be obtained by the dissolution of GeO_2_ from the Ge/GeO_2_ composite because a basic aqueous solution of tetrabutylammonium hydroxide (TBA^+^OH^−^) for the exfoliation of layered RuO_2_ could dissolve GeO_2_ by an acid–base reaction [24,25]. Subsequent thermal treatments transformed the layered RuO_2_ into a rutile form, which is thermodynamically stable and has a metal ion-permeable channel that enhances the Li-ion’s conductivity [26,27]. Therefore, the Ge-dispersed rutile-structured RuO_2_ can contribute to enhancing electrochemical performances through a combination of inherent Li-storage capacities and structural advantages. Therefore, the developed strategy provides a new approach for synthesizing advanced anode materials comprising rutile-structured RuO_2_-based Ge composites.

## 2. Results and Discussion

**Figure 2:**
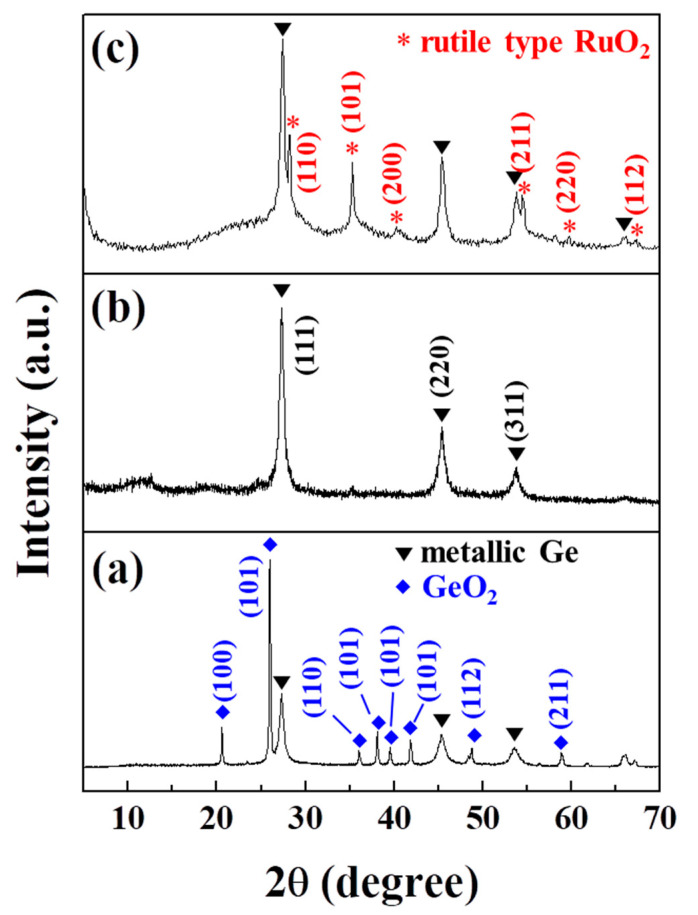
XRD patterns of (**a**) Ge/GeO_2_ NPs, (**b**) as-prepared Ge/RuO_2_, and (**c**) Ge/RuO_2_ nanocomposite.

XRD analyses were used to characterize the crystal structures of the samples during the reactions (Figure 2). The XRD pattern of the Ge/GeO_2_ NPs displays characteristic peaks of diamond-type cubic Ge and hexagonal GeO_2_ structures (Figure 2a). The XRD peaks assigned to GeO_2_ were not observed in the XRD pattern of the as-prepared Ge/RuO_2_ (Figure 2b), suggesting that the basic TBA^+^ solution containing exfoliated RuO_2_ dissolved GeO_2_ from the Ge/GeO_2_ NPs. Subsequent thermal treatments at 450 °C for 2 h resulted in the appearance of sharp peaks at 28.2, 35.3, 40.3, 54.4, 59.7, and 67.3°, corresponding to the (110), (101), (200), (211), (220), and (112) planes of rutile RuO_2_, respectively (Figure 2c). Therefore, reassembled RuO_2_ was transformed into rutile-type RuO_2_. Despite the thermal treatment, the crystal structure of the metallic Ge was still maintained without a decrease in the relative intensity of the peaks. These results indicate that Ge nanoparticles were successfully incorporated into the matrices of rutile-type RuO_2_.

**Figure 3:**
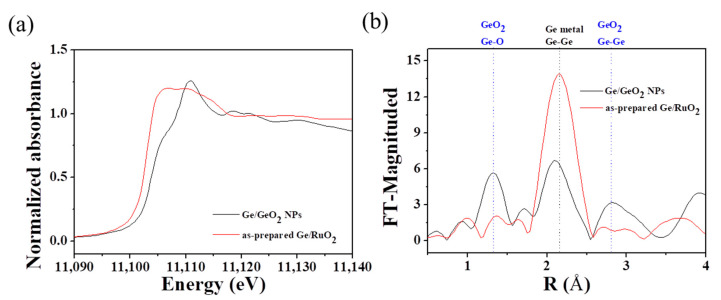
(**a**) Normalized XANES spectra acquired at Ge K-edge and (**b**) Fourier-transform of the EXAFS spectra of Ge/GeO_2_ NPs and as-prepared Ge/RuO_2_.

X-ray absorption spectral (XAS) analysis at the Ge K-edge was used to confirm the absence of GeO_2_ in the as-prepared Ge/RuO_2_ (Figure 3). The normalized XANES spectrum of the Ge/GeO_2_ NPs developed a shoulder peak around 11,105 eV and an edge peak around 11,110 eV, corresponding to the absorption peaks of Ge^0^ and Ge^4+^, respectively (Figure 3a) [28]. However, the absorption peak of Ge^4+^ was not observed in the XANES spectrum of as-prepared Ge/RuO_2_. Furthermore, Fourier transforms (FTs) of the k^3^-weighted EXAFS spectra of Ge/GeO_2_ NPs and as-prepared Ge/RuO_2_ were performed to determine the local structural variation around Ge ions (Figure 3b). The first and third FT peaks around 1.3 Å and 2.8 Å (non-phase-shift-corrected) are assigned to the (Ge-O) and (Ge-Ge) bonds of GeO_2_, whereas the second FT peak around 2.2 Å is assigned to the (Ge-Ge) bond of metallic Ge [28]. The peaks of GeO_2_ around 1.3 Å and 2.8 Å are clearly absent in the spectrum of as-prepared Ge/RuO_2_. Therefore, these results reveal that metallic Ge nanoparticles were included within RuO_2_, which is consistent with XRD results.

**Figure 4:**
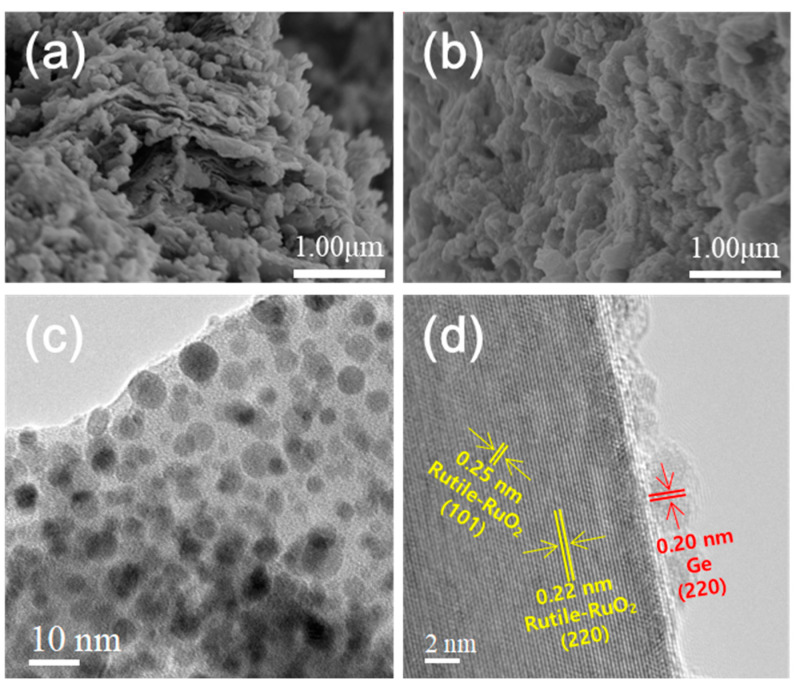
SEM images of (**a**) Ge/RuO_2_ and (**b**) Ge/RuO_2_ nanocomposite. TEM images of Ge/RuO_2_ nanocomposite in (**c**) low magnification and (**d**) high magnification.

The SEM images in Figure 4a,b, were used to analyze the morphology of the as-prepared Ge/RuO_2_ and Ge/RuO_2_ nanocomposite. These SEM images show that the planar morphology of the as-prepared Ge/RuO_2_ collapsed after thermal treatment, which means that layered RuO_2_ was transformed into rutile-type RuO_2_. The TEM images of the Ge/RuO_2_ nanocomposite show well-dispersed Ge nanoparticles on the rutile-type RuO_2_ (Figure 4c). Furthermore, the lattice fringes at 0.22, 0.25, and 0.20 nm are attributed to the (220) and (101) planes of rutile-type RuO_2_ and the (220) plane of metallic Ge, respectively (Figure 4d). These results are in good agreement with the XRD pattern of the Ge/RuO_2_ nanocomposite.

**Figure 5:**
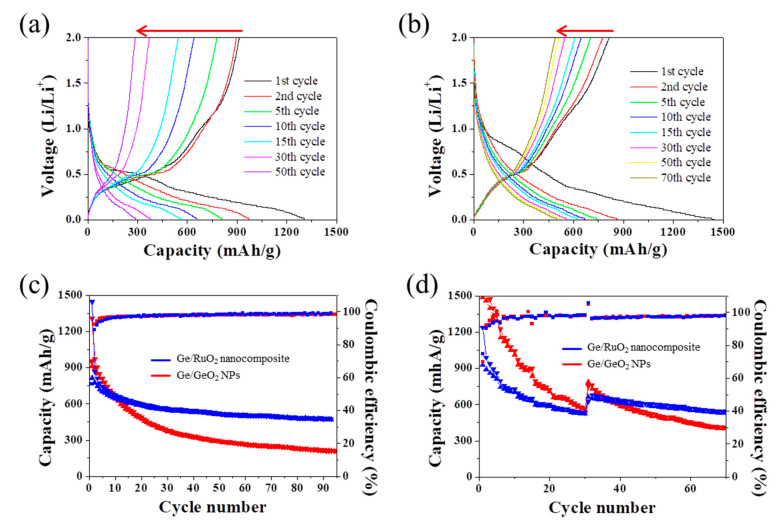
Electrochemical charge/discharge profile of (**a**) Ge/GeO_2_ NPs and (**b**) Ge/RuO_2_ nanocomposite at a current density of 100 mA g^−^^1^ (The red arrows indicate a decrease in capacity by cycling). Capacity and coulombic efficiency of Ge/GeO_2_ NPs and Ge/RuO_2_ nanocomposite (**c**) at a current density of 100 mA g^−^^1^ and (**d**) at various current densities from 0.1 mA g^−^^1^ to 1.0 mA g^−^^1^.

Figure 5a–c show the galvanostatic charge/discharge curves of the Ge/GeO_2_ NPs and Ge/RuO_2_ nanocomposite versus Li at a current density of 100 mA g^−1^ in the range of 0.01 and 2 V. The initial discharge/charge capacities of the Ge/GeO_2_ NPs and Ge/RuO_2_ nanocomposite were 1309/913 mA h g^−^^1^ and 1447/814 mA h g^−^^1^, corresponding to initial coulombic efficiencies of 69.7% and 56.2%, respectively. The initial irreversible capacity is derived from the formation of the solid electrolyte interphase (SEI). Nevertheless, the average CE value of the Ge/RuO_2_ nanocomposite after the first cycle was 97.7%, which is slightly higher than that of Ge/GeO_2_ NPs (97.6%). As a result, the discharge capacity of the Ge/RuO_2_ nanocomposite after the 90th cycle was 471 mA h g^−^^1^, which is significantly higher than that of the Ge/GeO_2_ NPs (211 mA h g^−^^1^). Figure 5d shows the rate capability of the Ge/GeO_2_ NPs and Ge/RuO_2_ nanocomposite at different current densities of 100, 300, 500, 700, 900, and 1000 mA/g for five successive cycles. After applying the current density of 1000 mA/g, the current density was reverted to the 100 mA/g. After the current density returned to 100 mA/g, the capacity retention of the Ge/RuO_2_ nanocomposite was 88%, whereas that of the Ge/GeO_2_ NPs was 58%. Therefore, the results indicate that rutile-type RuO_2_ prevents large volume changes, which contributes to not only good cycle stability, but also the capacity retention of anode materials.

**Figure 6:**
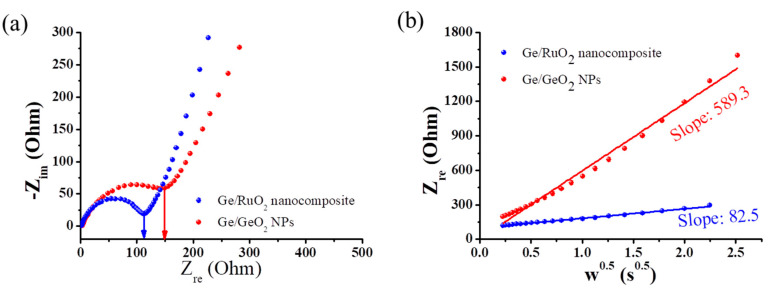
(**a**) Nyquist plot and (**b**) Warburg plot of Ge/GeO_2_ NPs and Ge/RuO_2_ nanocomposite.

As shown in Figure 6, EIS analysis was used to determine the electrochemical properties of the Ge/GeO_2_ NPs and Ge/RuO_2_ nanocomposite. The semicircle in the Nyquist plot is related to charge-transfer resistances, whereas the straight line is related to Li-ion diffusion (Figure 6a). At high frequencies, the radius of the semicircle of the Ge/RuO_2_ nanocomposite is much smaller than that of the Ge/GeO_2_ NPs. In other words, the charge-transfer impedance (R_ct_) of the Ge/RuO_2_ nanocomposite is significantly lower than that of Ge/GeO_2_ NPs, which means that the Ge/RuO_2_ nanocomposite has good electrical conductivity when compared with Ge/GeO_2_ NPs. These results indicate that rutile-type RuO_2_ facilitates charge transfer and enhances electrical conductivities. At low frequency, straight lines are observed in the Warburg plots, where the slope indicates the ionic conductivity (Figure 6b). The slope of the plot for the Ge/RuO_2_ nanocomposite (82.5) corresponded to a lower Warburg coefficient than that of the Ge/GeO_2_ NPs (589.3). This result demonstrates that the Ge/RuO_2_ nanocomposite has good Li-ion conductivity. Furthermore, the Warburg coefficient of the Ge/RuO_2_ nanocomposite was lower than that of the as-prepared Ge/RuO_2_ (Figure S1). This phenomenon indicates that the combination of well-dispersed Ge and rutile-type RuO_2_ with an ion-permeable channel contributes to higher Li-ion conductivities compared with Ge/GeO_2_ NPs and as-prepared Ge/RuO_2_.

**Figure 7:**
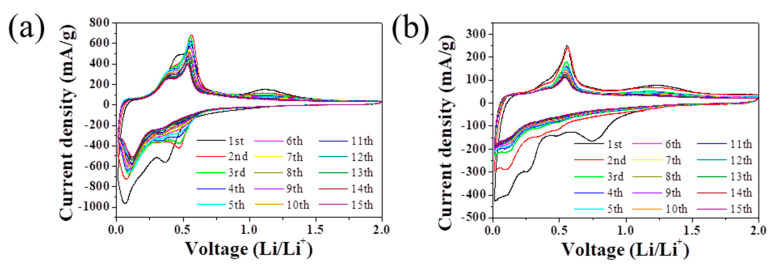
CV curves of (**a**) Ge/GeO_2_ NPs and (**b**) Ge/RuO_2_ nanocomposite at a scan rate of 0.1 mV s^−^^1^.

To understand the electrochemical process of Li-storage by the Ge/GeO_2_ NPs and Ge/RuO_2_ nanocomposite, CV data were acquired at a scan rate of 0.1 mV s^−^^1^ in the range of 0.01–2.0 V (Figure 7). The broad peak at 0.74 V in the first cathodic scan for the Ge/RuO_2_ nanocomposite is assigned to the formation of an SEI and Li_x_RuO_x_ [29]. Under 0.5 V, alloying peaks were observed for both samples. In the following anodic scan, the peaks in the range of 0.4–0.6 V and at 1.2 V indicate de-alloying and the reoxidation of Ge, respectively [10,19]. The overall CV profiles of the Ge/RuO_2_ nanocomposite are similar to those of Ge/GeO_2_ NPs, indicating that most of lithiation/delithiation processes occur on Ge nanoparticles.

**Figure 8:**
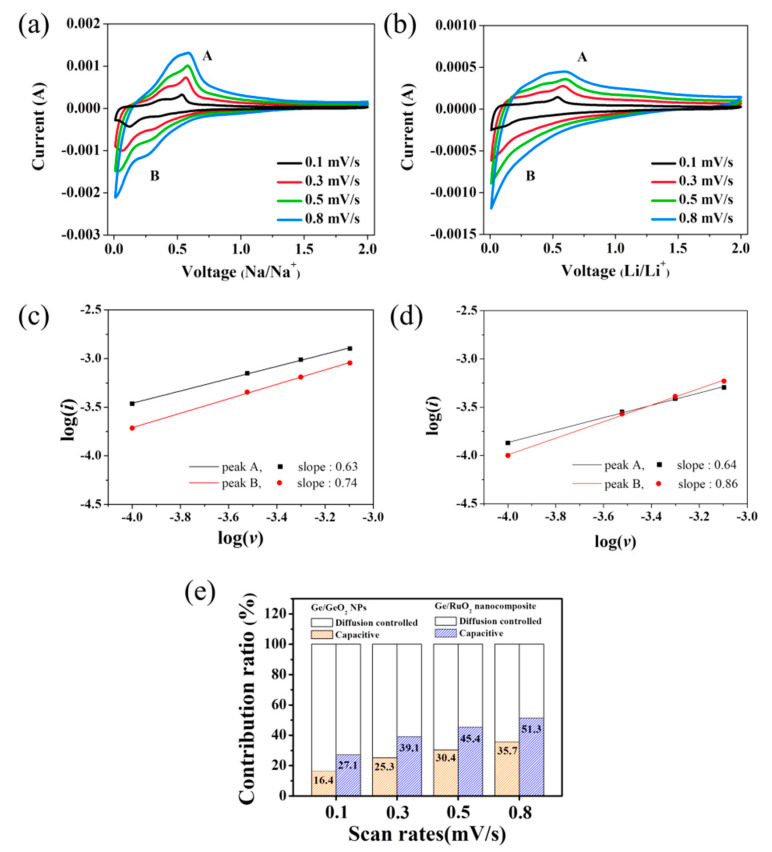
Contribution of diffusion-controlled and pseudocapacitive charge-storage processes in functioning cells at a scan rate of 0.8 mV s^1^ for (**a**) Ge/GeO_2_ NPs and (**b**) the Ge/RuO_2_ nanocomposite. log(*i*) versus log(*v*) plot of (**c**) Ge/GeO_2_ NPs and (**d**) Ge/RuO_2_ nanocomposite (The slopes were calculated from the peak A and B in Figure 8a,b). (**e**) Change in the contribution ratio of diffusion-controlled and pseudocapacitive charge-storage processes at varying scan rates.

For detailed electrochemical analysis, CV data were acquired at scan rates ranging from 0.1 to 0.8 mV s^−^^1^ (Figure 8a,b). The current is related to the scan speed, as expressed by Equations (1) and (2):

*i* = *av*^*b*^ (1)

log(*i*) = *b* log(*v*) + log(*a*) (2)

where *a* and *b* are adjustable parameters. *b*-values describe electrochemical behavior, such as diffusion-controlled and pseudocapacitive behavior [30]. Figure 8c,d show the log(*i*) versus log(*v*) plot, where the slope indicates the *b*-value. The *b*-value for the Ge/RuO_2_ nanocomposite was higher than that of the Ge/GeO_2_ NPs, indicating that the contribution of the pseudocapacitive process of the Ge/RuO_2_ nanocomposite was higher than that of Ge/GeO_2_ NPs. Figure 7b shows the capacitive contribution at scan rates of 0.1, 0.3, 0.5, and 0.8 mV s^−1^, respectively. The percentage of pseudocapacitive contribution increased with increasing scan speeds. The overall contribution ratios of the pseudocapacitance in the Ge/RuO_2_ nanocomposite are higher than those of the Ge/GeO_2_ NPs, which indicates that the Ge/RuO_2_ nanocomposite’s grain boundaries provide additional active sites for surface Li storage [31]. Therefore, these results reveal that the improved pseudocapacitive property of the Ge/RuO_2_ nanocomposite contributes to good cycle stability and enhances the rate performance of the electrode [32,33,34]. In conclusion, we determined that the synergic performance of rutile RuO_2_ and well-dispersed Ge NPs have key roles in the enhancement of ion conductivity, cyclability, and retention ability of the Ge/RuO_2_ nanocomposite.

## 3. Materials and Methods

### 3.1. Material

Potassium carbonate (K_2_CO_3_), ruthenium dioxide (RuO_2_), and tetrabutylammonium hydroxide (TBA^+^OH^−^) solutions (~40 wt% in water) were purchased from Sigma-Aldrich Corporation (St. Louis, MO, USA). Hydrochloric acid (HCl, 35–37%) was acquired from Duksan Pure Chemical Co., Ltd. (Ansan, Korea). Ethylamine (70% in water) was obtained from Junsei Chemical Co., Ltd. (Tokyo, Japan). Ge/GeO_2_ powder (99.9% purity, 35 nm) was purchased from RNDKOREA (Gwangmyeong, Korea).

### 3.2. Preparation of Ge/GeO_2_ NPs

Commercially available Ge/GeO_2_ nanoparticles (NPs) were ground in a mortar for 30 min to obtain a fine powder. The obtained powder (0.3 g) was added to a mixture of 15 mL distilled water and 15 mL acetone, followed by ultrasonication for 30 min (3 s on followed by 1 s off) to obtain a colloidal suspension of Ge/GeO_2_ NPs.

### 3.3. Synthesis of the Exfoliated RuO_2_ NSs

A colloidal suspension of exfoliated RuO_2_ nanosheets (NSs) was synthesized according to the procedure shown in Figure 1. Layered potassium ruthenate (K_0.2_RuO_2.1_·nH_2_O) was synthesized using a solid-state reaction. In a mortar, potassium carbonate (K_2_CO_3_) was mixed with ruthenium dioxide (RuO_2_) in a molar ratio of 5:8. This mixture was pelletized and calcined for 12 h at 850 °C under Ar atmosphere. The obtained sample was washed with distilled water to remove water-soluble impurities. Potassium ruthenate was subjected to a proton exchange reaction by exchanging potassium ions with protons in the ruthenate layer in 1 M HCl aqueous solution at 60 °C for 72 h. During this proton exchange reaction, the 1 M HCl aqueous solution was replaced with a fresh batch every 24 h. The layered protonic ruthenate (H_0.2_RuO_2.1_·nH_2_O) was also subjected to ion-exchange reaction in 50% aqueous ethylamine (EA) solution at room temperature for 24 h to obtain ethylammonium (EA^+^)-intercalated ruthenate. The EA^+^-intercalated ruthenate was collected by centrifugation, washed with distilled water, and then reacted with a 10% tetrabutylammonium hydroxide (TBA^+^OH^−^) aqueous solution for 120 h. Thus, exfoliated RuO_2_ NSs stably suspended in an aqueous solution of tetrabutylammonium hydroxide were prepared.

### 3.4. Synthesis of Ge/RuO_2_ Nanocomposites

Ge/RuO_2_ nanocomposites were prepared according to the procedure shown in Figure 1. A colloidal suspension of RuO_2_ NSs (4 g/L) and colloidal suspension of Ge/GeO_2_ nanoparticles (10 g/L) were mixed in a flask at a mass ratio of 3:7, and the mixture was stirred for 3 h. From this synthesis process, GeO_2_ NPs were dissolved in a basic aqueous solution of tetrabutylammonium hydroxide (TBA^+^OH^−^). The mixture was collected via centrifugation (15,000 rpm for 5 min) to obtain as-prepared Ge/RuO_2_ nanocomposites. The as-prepared Ge/RuO_2_ nanocomposites were dried at room temperature and then heat-treated at 450 °C for 2 h. In this synthesis process, RuO_2_ NSs were crystallized to the rutile phase, and Ge/RuO_2_ nanocomposites were obtained, in which Ge nanoparticles were homogeneously distributed onto the matrices of RuO_2_.

### 3.5. Structural Characterization

The crystalline phases of the samples were characterized by powder X-ray diffraction (PXRD; Bruker D2 Phaser, Billerica, MA, USA) with Cu-Kα radiation (λ = 1.54056 Å). The morphologies and structures of the products were characterized by field-emission scanning electron microscopy (FE-SEM, Hitachi SU8220, HITACHI, Japan) and field-emission transmission electron microscopy (FE-TEM, Titan G2 ChemiSTEM Cs Probe, FEI Company, Eindhoven, the Netherlands). X-ray absorption fine structure (XAFS) spectra of the powder samples were obtained using the 8C NanoProbe XAFS beamline (BL8C) at the Pohang Accelerator Laboratory (PAL, Pohang, Korea). XAFS spectra of the powder samples were collected in transmission mode at room temperature. The collected XAFS spectra were analyzed using the IFEFFIT software package. IFEFFIT’s project pages and information are at SourceForge.net, http://sourceforge.net/projects/ifefit/, and http://cars.uchicago.edu/ifefit (accessed on 5 September 2022) [35,36].

### 3.6. Electrochemical Characterization

The anode material was prepared by mixing the synthesized active material, conductive carbon (Super P), and a binder (polyacrylic acid) in NMP solvent in a weight ratio of 7:2:1. The slurry of anode material was deposited on the copper current collector at a mass loading of approximately 1.5 mg cm^−2^ using the doctor blade method. This electrode was dried in an oven at 60 °C for 5 h (under ambient atmosphere) and dried again in a vacuum oven at 100 °C for 12 h. Subsequently, this active electrode was transferred to a glovebox filled with Ar. The CR2032 coin-type cell for the electrochemical experiment was assembled in a glovebox filled with high-purity argon. The prepared anode and Li metal counter electrodes were electronically separated using a 3501-type separator (Celgard 3501). A solution of LiPF_6_ (1 M) in propylene carbonate/fluoroethylene carbonate (98:2, *w*/*w*) was used as the electrolyte. Galvanostatic charge/discharge tests were performed using a battery tester (Maccor K4300, Tulsa, OK, USA) in a potential window of 0.01–2.0 V versus Li^+^/Li. Cyclic voltammetry (CV) measurements were performed using a multi-channel potentiostat (WonATech WMPG1000, Seoul, Korea) in the potential window of 0.01–2.0 V versus Li^+^/Li. Electrochemical impedance spectroscopy (EIS) measurements were conducted using a single-channel potentiostat (WonATech ZIVE SP2, Seoul, Korea) in the frequency range 0.01 Hz to 100 kHz at open-circuit voltage. All electrochemical measurements were performed at room temperature.

## 4. Conclusions

A rutile-type RuO_2_-based Ge nanocomposite was successfully obtained via successive exfoliation/reassembly and heat-treatment processes. The structural characterization of the samples revealed that metallic Ge nanoparticles were well-dispersed within the matrices of rutile-type RuO_2_. The rutile structure of RuO_2_ contributes to good charge transfer and high ionic conductivity. Furthermore, well-dispersed Ge nanoparticles formed grain boundaries with rutile RuO_2_, enhancing pseudocapacitive properties. Consequently, the Ge/RuO_2_ nanocomposite exhibits excellent electrochemical properties for LIBs, with good cycling stability and capacity retention.

## Data Availability

The data presented in this study are available upon request from the corresponding author.

## Supplementary Materials

The following supporting information can be downloaded at: https://www.mdpi.com/article/10.3390/ijms231911766/s1.
