# Supplementary material for: Exfoliation and Reassembly Routes to a Ge/RuO2 Nanocomposite as an Anode for Advanced Lithium-Ion Batteries
Source: Int J Mol Sci. 2022 Oct 4;23(19):11766. doi: 10.3390/ijms231911766 (PMC9569558; doi:10.3390/ijms231911766)
Supplement: Supplementary file 1 [file ijms-23-11766-s001.zip › ijms-1929799-supplementary.pdf]

Exfoliation and Reassembly Route to a Ge/RuO_2 Nanocomposite as an Anode for Advanced Lithium-Ion Batteries

Jeong-Hun Jang,† Minseop Lee,† Ji-Hye Koo† and Seung-Min Paek*

Department of Chemistry, Kyungpook National University, Daegu 41566, Korea

Supporting Information Appendix

This Document contains supplementary data as referred to in the main manuscript.

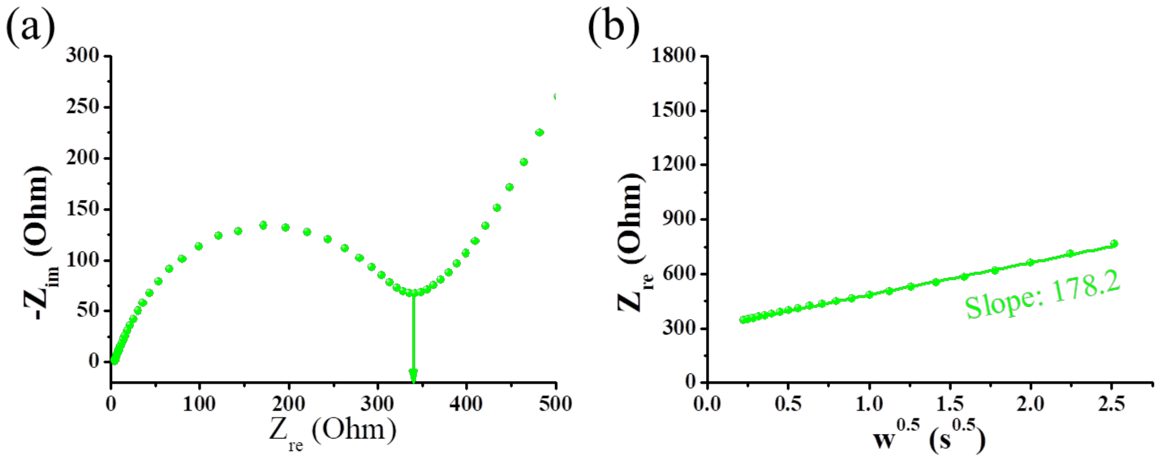

Figure S1. (**a**) Nyquist plot and (**b**) Warburg plot of as-prepared Ge/RuO_2.
